# DJ1 expression downregulates in neuroblastoma cells (SK-N-MC) chronically exposed to HIV-1 and cocaine

**DOI:** 10.3389/fmicb.2015.00749

**Published:** 2015-07-28

**Authors:** Upal Roy, Venkata S. R. Atluri, Marisela Agudelo, Adriana Yndart, Zaohua Huang, Madhavan Nair

**Affiliations:** Department of Immunology, Institute of NeuroImmune Pharmacology, Centre for Personalized Nanomedicine, Herbert Wertheim College of Medicine, Florida International University, Miami, FL, USA

**Keywords:** HIV-1, HIV-associated neurological disorder, cocaine, DJ1, Parkinson’s disease, SK-N-MC

## Abstract

**Background:** HIV-associated neurological disorder (HAND) has long been recognized as a consequence of human immunodeficiency virus (HIV) infection in the brain. The pathology of HAND gets more complicated with the recreational drug use such as cocaine. Recent studies have suggested multiple genetic influences involved in the pathology of addiction and HAND but only a fraction of the entire genetic risk has been investigated so far. In this regard, role of DJ1 protein (a gene linked to autosomal recessive early-onset Parkinson’s disease) in regulating dopamine (DA) transmission and reactive oxygen species (ROS) production in neuronal cells will be worth investigating in HIV-1 and cocaine exposed microenvironment. Being a very abundant protein in the brain, DJ1 could serve as a potential marker for early detection of HIV-1 and/or cocaine related neurological disorder.

**Methods:** *In vitro* analysis was done to observe the effect of HIV-1 and/or cocaine on DJ1 protein expression in neuroblastoma cells (SK-N-MC). Gene and protein expression analysis of DJ1 was done on the HIV infected and/or cocaine treated SK-N-MC and compared to untreated cells using real time PCR, Western Blot and flow cytometry. Effect of DJ1 dysregulation on oxidative stress was analyzed by measuring ROS production in these cells.

**Results:** Gene expression and protein analysis indicated that there was a significant decrease in DJ1 expression in SK-N-MC chronically exposed to HIV-1 and/or cocaine which is inversely proportional to ROS production.

**Conclusion:** This is the first study to establish that DJ1 expression level in the neuronal cells significantly decreased in presence of HIV-1 and/or cocaine indicating oxidative stress level of DA neurons.

## Introduction

The introduction of antiretroviral therapy (ART) has significantly improved the morbidity and mortality of human immunodeficiency virus (HIV) infected patients; however, the virus still persists in reservoirs like the brain even in ART treated patients (Best et al., 2009; Varatharajan and Thomas, 2009; Kanmogne et al., 2012). One of the major neurological disorders that are most frequently observed in patients with ART therapy is HIV-associated neurological disorder (HAND; Everall et al., 2009; Roy et al., 2013). The molecular mechanisms of cognitive impairment during HIV infection are still unclear, although previous studies have indicated HIV-associated inflammation and neurodegenerative pathology in the brain of HAND patients (Grant, 2008; Robinson-Papp et al., 2008). Few studies have assessed the genetic risk factors that are associated with cognitive impairment among HIV-1 infected patients although there is no clear clinical correlation (Payton, 2006). There are some accumulating evidences suggesting that HIV-1 and ART may facilitate the development of neurodegenerative disease like Parkinson disease (PD) via many signaling mechanisms including but not limited to inflammation, mitochondrial dysfunction, and interference with ubiquitin proteasome pathway (Brew et al., 2009; Tisch and Brew, 2009; Wilhelmus et al., 2012). Persistent low levels of HIV replication in presence of ART might predispose host to neurodegeneration through low level of neuro inflammation (Brew et al., 2009). Several groups have reported increasing incidences of HIV related to cognitive impairment among virally suppressed HIV patients who are on ART for a longer period of time (Cysique et al., 2006; Tisch and Brew, 2009). This phenomenon also poses an increasing challenge on identifying or treating HIV infected patients with neurological disorders. HAND possesses many distinct neurological disorders including cognitive impairment, behavioral abnormalities and motor disorders which is also very common in PD (Price, 1996; Cysique et al., 2006). Both PD and HIV infection have a common anatomical target in the brain called substantia nigra and a common neurochemical disturbance called dopamine (DA) deficiency (Brew et al., 2009; Gago et al., 2011).

The DA deregulation was also observed in cocaine addicted patients who are infected with HIV makes this clinical scenario relatively more complex (Nair et al., 2005). A permanent imbalance in DA level in the brain due to chronic cocaine addiction can also add one another layer of complexity in detecting neurodegenerative diseases especially during HIV infection (Meade et al., 2015). Thus, the key factor that regulates all these functions will be of prime importance to understand this highly complicated disease pathology. In order to address this issue, we have investigated DJ1, a protein that is abundant in the brain and also a key regulator of DA transmission and reactive oxygen species (ROS) balance (Blesa and Przedborski, 2014; Lopert and Patel, 2014; Sun et al., 2014).

DJ-1 (PARK7) is a neuroprotective protein that protects cells from oxidative stress (Lopert and Patel, 2014; Sun et al., 2014). Initially, DJ-1 gene was linked to autosomal recessive early-onset of PD. It is a homodimeric protein belonging to the Thi/Pfp1 super family, highly conserved in most living things from human beings to *Escherichia coli* and is localized in the cytoplasm, nucleus, and mitochondria (Requejo-Aguilar et al., 2015). Although its normal physiological function is not entirely known, it has been shown to be involved in DA neurotransmission, particularly through the dopamine transporter (DAT). It normally inhibits reuptake of DA through DAT which helps in maintaining DA levels in the brain. The protective role of DJ-1 against oxidative stress is well known in PD (Hering et al., 2004; Liang et al., 2007). DJ1 protects neurons from oxidative stress by scavenging H_2_O_2_ from neuronal environment and thus it also protects mitochondrial integrity in these cells (Schapira, 2009; Vasseur et al., 2009; Wilhelmus et al., 2012). Most importantly, DJ-1 is ubiquitously expressed throughout the body including the brain (Schulte and Gasser, 2011). All these observations explain that DJ1 tries to regulate DA transmission in the brain which could get deregulated with the addiction of drugs like cocaine. At the same time, DJ1 also regulates oxidative stress which is highly affected by HIV infection in the brain (Brew et al., 2009). Considering these two important factors, it is crucial to investigate if DJ1 has any role in controlling neuronal functioning and homeostasis during HIV infection and cocaine treatment. In the present study, we have demonstrated a direct effect of HIV-1 and/or cocaine on DJ1/PARK7 expression and function that may be associated with neuronal damage and associated disorders. This study will potentially increase our understanding of the role of DJ1 in neuronal dysregulation during HIV-1 infection and cocaine abuse.

## Materials and Methods

### Cell Culture and Reagents

Human neuroblastoma SK-N-MC cells were obtained from ATCC (ATCC Cat # HTB-10) and cultured in Eagle’s minimum essential medium (MEM; catalog # 30-2003) supplemented with fetal bovine serum to a final concentration of 10% (catalog # 30-2020) and 1% antibiotic/antimycotic solution (Sigma-Aldrich, St. Louis, MO, USA). HIV-1Ba-L (clade B) (NIH AIDS Reagent Program Cat. # 510) was obtained through AIDS Research and Reference Reagent Program, Division of AIDS, NIAID, NIH. Park7 primer was obtained from life technologies, NY, USA (Cat. # 4331182, Hs00994896_g1). DJ1 antibody (#2134) was purchased from Cell Signaling Technology, Inc, USA. Cocaine hydrochloride was obtained from Sigma (Cat# C5776).

### HIV-1 Exposure and Cocaine Treatment of SK-N-MC Cells

SK-N-MC cells were exposed to HIV-1 using the previously described protocol (Ohagen et al., 1999; Eugenin and Berman, 2007; Atluri et al., 2013, 2014; Kurapati et al., 2013) with slight modifications. Briefly, SK-N-MC (1 × 10^6^ cells) cells were cultured overnight in T-75 flasks using MEM. SK-N-MC cells were initially exposed to three different concentrations of HIV-1 (50, 100, and 300 ng) in order to observe the effect of DJ1 gene expression in presence of HIV. After 24 h of HIV exposure, cell pellet was collected for RT-PCR analysis. Based on dose of HIV that showed highest expression of DJ1, further experiments were designed. In parallel experiment, increasing doses of cocaine (10, 100, 1000, and 3000 nM) were used to treat SK-N-MC cells for 24 h as per previous established protocol (Gandhi et al., 2010). At the end of incubation, cells were used for RT-PCR analysis as mentioned above. A graphical representation was made with respect to fold change of gene expression in response to different concentration of cocaine. Further experiments were designed with the cocaine concentration that showed highest response to DJ1 gene expression.

Finally, SK-N-MC cells were exposed to pre-optimized doses of HIV-1 and cocaine separately and in combination for 24 and 72 h, respectively. In case of co-incubation, HIV-1 and cocaine were added to the cells simultaneously at once and no fresh media was added during the entire period of incubation in order to maintain the concentration. At the end of incubation, cells were harvested for RT-PCR, Western blot and flow-cytometric analysis at 72 h post incubation.

In order to observe the viral replication in SK-N-MC, cells were exposed to a pre-optimized concentration of HIV-1 as per published protocol with a little modification (Harouse et al., 1995; Alvarez Losada et al., 2002; Atluri et al., 2014). Initially, SKN-MC were exposed to HIV-1 for 12 h. After incubation, cells were washed with phosphate buffer (PBS) twice to remove any unattached virus and incubated for 5 days with fresh media. Every 48 h, half of the medium was replaced with the fresh medium and the supernatant obtained from the used medium was used for the p24 antigen estimation using ELISA kit (ZeptoMetrix Corp. Cat # 0801200). Control (unexposed) cells were included in the set-up of all experiments. Viral replication was measured in cell supernatant with respect to p24 level at different time points (day 1, 3, and 5) and graphical representation was made for p24 (pg/ml) vs. days of incubation.

### Quantitative Real Time PCR

Gene expression was quantitated using real time PCR. Upon desired treatment period, cells were harvested, RNA from cell pellets was extracted using RNeasy mini kit (Qiagen, GmbH, Germany) followed by cDNA synthesis using high capacity reverse transcriptase cDNA kit (Applied Biosystems) to perform quantitative real time PCR (qRT-PCR) using Taqman gene expression assays (Applied Biosystems) for DJ1 (Hs00994896_g1) and GAPDH (Hs99999905_m1). GAPDH was served as an internal control. Relative abundance of each mRNA species was assessed using brilliant Q-PCR master mix from Stratagene with Mx3000P instrument which detects and plots the increase in fluorescence vs. PCR cycle number to produce a continuous measure of PCR amplification. Relative mRNA species expression was quantitated and the mean fold change in expression of DJ1 gene was calculated using the comparative CT method (Transcript Accumulation Index, TAI = 2-ΔΔCT). All data were normalized with an endogenous reference gene, GAPDH. In addition, results on RNA from treated samples were normalized with control (untreated sample).

### DJ1 Protein Levels by Western Blot Analysis:

For SDS-PAGE, similar amounts of control and test group SKN-MC cellular protein, typically 40 μg per lane were used. All the protein samples were separated by using Any KD Mini-Protean TGX precast Gels (Bio-Rad, Cat # 456–9034). Proteins were transferred to nitrocellulose membranes, and the quality of protein measurement, electrophoresis, and transfer was checked by staining with Ponceau S. Membranes were blocked with 5% skimmed milk in TBS-T (20 mM Tris buffer, pH 7.5, 0.5 M NaCl, 0.1% Tween 20) for 1 h at room temperature and incubated at 4°C overnight in the anti-DJ1 primary antibody (Cell signaling, cat # 2134) 1/1000 diluted in 2% skimmed milk in TBS-T. Subsequently, blots were washed in TBS-T (four times, 10 min each) and incubated for 1 h at room temperature in horse radish peroxidasegoat anti-rabbit antibody (Promega, Cat # W401B) diluted 1/2500 in 2% skim milk in TBS-T. After additional washings, protein bands were detected by chemiluminiscence using SuperSignal West Pico Luminol/Enhancer (Thermo Scientific, Cat # 1856136) and SuperSignal West Pico substrate (Thermo Scientific, Cat # 1856135). ImageJ software was used to quantify the protein expression on the blot and expressed as a fold change with respect to treatment.

### Intracellular DJ1 Protein Analysis by Flow Cytometry

To assess the levels of DJ1 protein in SK-N-MC, the cells were treated with HIV-1 (100 ng) and/or cocaine (1 μM) for 72 h. The cells were then harvested and counted; equal number of cells (1 × 10^6^) were aliquoted in 12 mm × 75 mm polystyrene falcon tubes (catalog # 352058, BD Biosciences, San Jose, CA, USA), blocked with 1:1 ratio of human and normal goat serum (Chemicon International, Temecula, CA, USA), fixed and permeabilized with Cytofix/Cytoperm solution (BD Bioscience). The DJ1 protein was detected with primary monoclonal antibody, rabbit anti-DJ1 (Cell Signaling, CA, USA) and secondary antibody, FITC-conjugated goat anti-rabbit IgG antibody (Millipore). Cells were acquired on an Accuri C6 instrument (BD Accuri, Ann Arbor, MI, USA) and analyzed with FloJo software (Tree Star, INC, Ashland, OR, USA). A total of 10,000 events were collected for each sample. Cells were gated based on unlabeled and secondary antibody controls. Cells positive for specific protein are shown in the histogram overlay with shifted mean fluorescence intensity compared to controls.

### Effect of HIV-1 and/or Cocaine on ROS Production in SK-N-MC

Reactive oxygen species production following exposure to HIV-1 and/or cocaine in SK-N-MC were detected using dichlorofluorescein diacetate assay (DCF-DA; Molecular Probes, Eugene, OR, USA) as per published protocol (Agudelo et al., 2011). Cells were cultured in 96-well plates (100,000 cells/well) overnight to allow them to adhere on the surface of the well. The next day, cells were treated with optimized concentration of HIV-1 and/or cocaine for 24 and 72 h, respectively, without changing media during incubation. After incubation, cells were washed and treated with antioxidant, catalase (0.001 mg) for 2 h. Next, the cells were washed and treated with DCF-DA (100 μM) for 1 h at 37°C and finally read in a BioTek Synergy HT microplate reader (excitation 485 nm and emission 528 nm; BioTek, Winooski, VT, USA). Cells treated with H_2_O_2_ (50 μM) for 2 h was considered as positive control. Furthermore, fluorescence was visualized in an Olympus IX51 microscope (Olympus America Inc., Center Valley, PA, USA) and images captured and analyzed with the Qimaging camera and software (QImaging, British Columbia, Canada).

### Data Analysis

PCR analysis was performed at least three times and the values obtained were averaged. Protein analysis (Western Blot and flow cytometry) was done at least three times in order to obtain quantitative analysis of the protein blot and to measure the fold change in protein expression. In the western blot, proteins relative band density was measured by using ImageJ software. All the results were expressed as mean ± standard error of the mean. Statistical analysis of two groups was performed by Student’s *t*-test, while more than two groups were analyzed using one way ANOVA. Differences were considered significant at *p* ≤ 0.05. If the combined effect observed is significantly greater/less than the expected (additive) effect, it was considered synergism (Tallarida, 2011). Data analysis was performed with the Statistical Program, GraphPad Prism software (La Jolla, CA, USA).

## Results

### DJ1 Expression Increased in Presence of HIV-1

**FIGURE 1:**
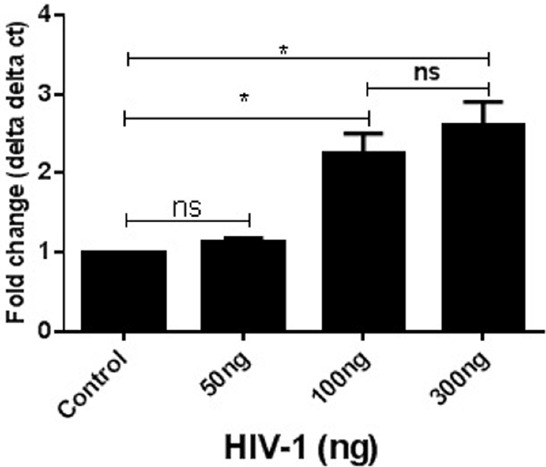
**Acute exposure of HIV-1 increased DJ1/PARK7 expression in SK-N-MC cells.** Different concentrations of HIV-1 were exposed (50–300 ng) to SK-N-MC cells for 24 h. RT-PCR data represented with respect to fold changes in gene expression compared to control. The fold change expression was found to be statistically significant (*p < 0.05).

SK-N-MC cells were exposed to three different concentrations of HIV-1 virus (10, 100, and 300 ng) for 24 h. At the end of the incubation cells were harvested and used for qRT-PCR analysis. Gene expression was measured with respect to the fold change compared to experimental control. GAPDH was kept as internal control for this experiment. The data indicated that with increasing concentrations of virus there was an increase in DJ1 expression up to 100 ng (*p* ≤ 0.05). However, increase in gene expression beyond that concentration was not significant (300 ng; Figure 1). Thus, 100 ng was considered to be optimum for further experimental analysis.

### Exposure of Cocaine Increased DJ1 Expression

**FIGURE 2:**
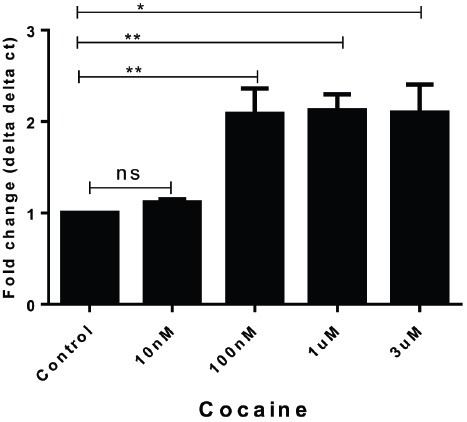
**Acute treatment of cocaine increased DJ1/PARK7 gene expression in SK-N-MC cells.** Different concentrations of cocaine (10 nM–3 μM) were exposed to SK-N-MC cells for 24 h and gene expression data were represented with respect to fold change compared to control. The gene expression data was statistically validated with unpaired *t*-test and statistical significance (*p* values) indicated in the order of **p < 0.05, *p < 0.005.

SK-N-MC cells were exposed to four different concentrations of cocaine (10, 100, 1000, and 3000 nM) for 24 h. At the end of incubation, cells were harvested for RT-PCR analysis. As mentioned above, the comparative gene expression analysis was done with respect to delta delta ct values of PCR data keeping GAPDH as internal control. Data represented in Figure 2 indicates that in 10 nM–3 μM of cocaine treatment there was significant increase of DJ1 gene expression compared to control within 24 h incubation (*p* < 0.001). In this regard, 1 μM concentration was considered as optimum concentration for further study.

### DJ1 Expression Decreased in Presence of HIV-1 and Cocaine

**FIGURE 3:**
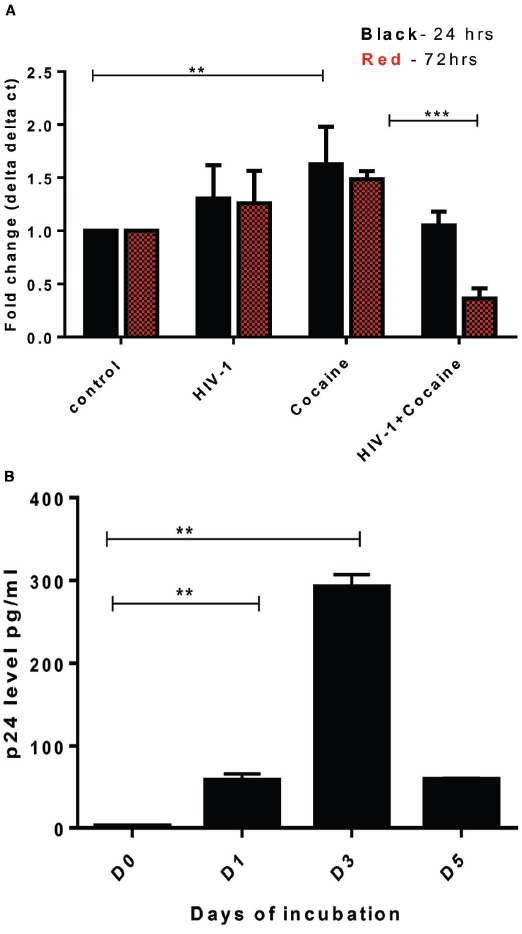
**Exposure of HIV-1 and cocaine significantly decreased DJ1/PARK7 expression in SK-N-MC cells. (A)** SK-N-MC cells were exposed to HIV-1 (100 ng), cocaine (1 μM) and HIV-1 with cocaine, respectively in order to observe the gene expression after 24 h (black) and 72 h (red) of incubation. RT-PCR data was represented with respect to fold change as compared to control. Statistical significance was calculated with unpaired *t*-test and *p* values were expressed in as **p = 0.009, ***p = 0.0006. **(B)** HIV-1 exposure to SK-N-MC indicated that there was significant increase of p24 level up to day 3 (72 h) indicating viral replication within these cells (**p < 0.05).

SK-N-MC cells were treated with cocaine (1 μM), HIV-1 (100 ng) separately and in combination for 24 and 72 h, respectively. Untreated SK-N-MC were used as control. After incubation, cells were washed out and cell pellets were collected for qRT-PCR analysis. Compared to untreated/uninfected control, HIV-1 or cocaine independently increased the DJ1 gene expression (Figure 3A). However, the combined effects of HIV-1 and cocaine significantly decreased the DJ1 expression especially 72 h post treatment (*p* = 0.0006). In order to observe the HIV replication in SK-N-MC cells, 100 ng of HIV-1 was exposed to these cells and p24 level were measured up to 5 days post infection as explained in method section. The p24 level data indicated that there was subsequent increase in viral replication up to 72 h (Figure 3B). Therefore, following experiments were done after incubation of 72 h.

### Exposure of HIV-1 and/or Cocaine Decreased DJ1 Protein (Western Blot)

**FIGURE 4:**
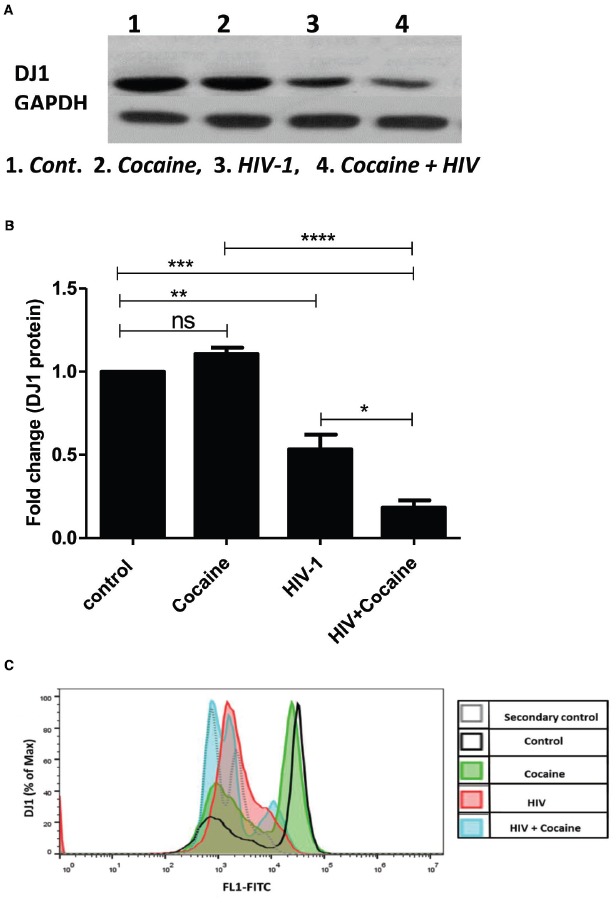
**Combined effect of HIV-1 and cocaine markedly decreases DJ1 protein expression in SK-N-MC cells as measured by western blot and flow cytometry. (A)** Protein profile of DJ1 gene in SK-N-MC exposed to HIV-1 (100 ng) and/or cocaine (1 μM). **(B)** Volumetric analysis of Western Blot data with respect to fold change compare to untreated cells (control). Significance values were represented as *p < 0.05, **p < 0.005, ***p < 0.0005, ****p < 0.0001. **(C)** Representative overlay histogram of the total cells stained with primary monoclonal antibody, rabbit anti-DJ1 and secondary antibody, FITC-conjugated goat anti-rabbit IgG. The light gray dotted line histograms represent the secondary antibody control; the black line histogram represents the untreated/uninfected control; the green histogram represent the cocaine treated cells; the red histogram represents the HIV infected cells; and the blue histogram represents the cocaine treated and HIV infected cells. 10,000 events were acquired. Data are representative of three independent experiments.

SK-N-MC cells were grown in T-75 culture flask and treated with HIV-1 (100 ng) and/or cocaine (1 μM), respectively, for 72 h. At the end of the incubation, the cells were harvested and cell pellets were collected from respective treatments for Western Blot analysis. Western blot was developed with the help of anti-human DJ1 antibody (Cell signaling, USA) and GAPDH was used as internal control (Figure 4A). The volumetric analysis of western blot indicated that DJ1 protein expression was significantly inhibited with the exposure of HIV-1. At the same time, the synergistic effects of HIV-1 and cocaine have further reduced the DJ1 expression in SK-N-MC cells which was significantly lower than untreated cells and HIV-1 exposed cells (*p* < 0.0005; Figure 4B). This observation strongly indicated that the HIV-1 and cocaine have dramatically inhibited the expression of DJ1 protein in SK-N-MC cells.

### Effect of HIV-1 and/or Cocaine on DJ1 Protein Expression on SK-N-MC Cells (Flow Analysis)

Flow cytometric analysis of intracellular DJ1 protein in HIV-1 and/or cocaine exposed cells correlated with the Western blot data. Intracellular levels of DJ1 decreased after 72 h exposure to HIV-1 and/or cocaine (Figure 4C). Furthermore, the combined effects of HIV-1 and cocaine caused a significant decrease in DJ1 expression compared to the individual treatment and control. Thus, the DJ1 down-regulation in SK-N-MC cells indicates a direct suppressive effect of cocaine and HIV.

### Exposure of HIV-1 and/or Cocaine Increases ROS Production in SK-N-MC Cells

**FIGURE 5:**
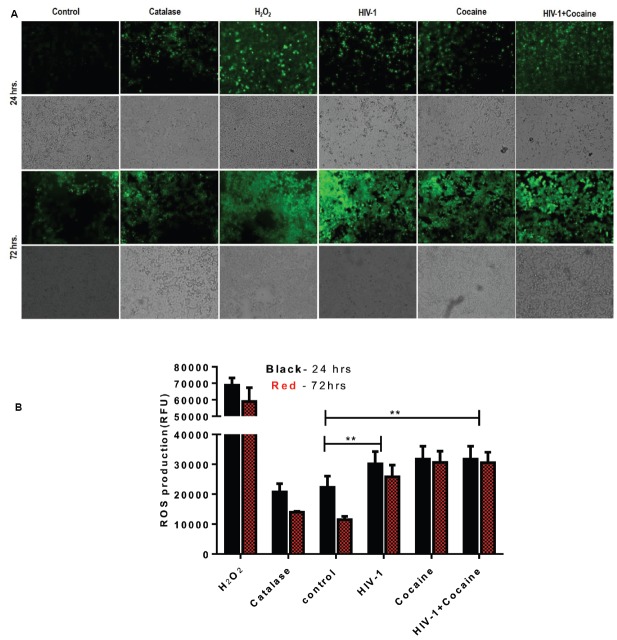
**HIV-1 and/or cocaine exposure enhanced ROS production in SK-N-MC cells after 24 and 72 h. (A)** HIV-1 and cocaine induced ROS production was observed in SK-N-MC. Cells were pretreated with H_2_O_2_, catalase, HIV-1, cocaine and HIV-1+cocaine, respectively. The fluorescence was detected at 485 excitation and at 528 emission spectra. Images were representative of four independent experiments. **(B)** SK-N-MC cells were exposed to predetermined concentration of HIV-1 and cocaine for 24 and 72 h, respectively. At the end of the incubation ROS production was measured in treated cells and compared with untreated control. H_2_O_2_ treatment was served as a positive control and Catalase as an antioxidant. Data are expressed as mean ± SE of relative fluorescence units (RFU) values of four independent experiments. The statistical significance were expressed as *p* values (**p < 0.0001).

DJ1 is one of the major regulator of ROS production in neuronal cells (Lopert and Patel, 2014). SK-N-MC cells were exposed to HIV-1 and/or cocaine for 24 and 72 h, respectively. At the end of the incubation, cells were used to perform ROS assay as per previously published protocol (Agudelo et al., 2011). In this study, we have observed an increase in the ROS productions after treatment with HIV-1 and/or cocaine, which indicates that there is a certain amount of ROS imbalance in SK-N-MC cells after cocaine treatment and HIV-1 exposure. Fluorescence images were captured with an Olympus IX51 microscope indicating the retention of oxidized DCF reagent in the cell. As per protocol, reduced form of DCF is non-fluorescent until it gets oxidized with the help of intracellular esterases of treated cells. Therefore, higher retention of oxidized-DCF in HIV-1, cocaine, and HIV-1+cocaine treated cells was indicative of ROS production compared to untreated cells. The H_2_O_2_ treated cells served as a positive control for ROS production (Figure 5A). The intracellular fluorescence of treated cells were detected by microplate reader and expressed as mean relative fluorescence units (RFU) with respect to different treatments as explained in Figure 5B. The comparative analysis of ROS production between 24 and 72 h did not show any significant difference in the HIV-1 and/or cocaine treated cells. Nonetheless, there was substantial increase in ROS production with exposure to HIV-1 or cocaine and in combination compared to untreated cells (*p* < 0.0001).

## Discussion

Neurocognitive impairment has long been recognized as a consequence of HIV-1 infection. Combination ART has altered the pathological manifestation of HIV to a great extent; however, virus still persists in the brain and can cause neuronal abnormalities. Therefore, it is very important to understand the molecular mechanisms of virus mediated neuronal defects. Ever since ART became widely available in developed countries, HIV associated dementia (HAD) has become quite rare, observed in only 2–3% of patients in research studies. However, the subtler forms of HAND remain frequent and become more likely with increasing age (Devlin et al., 2012; Hargus and Thayer, 2013). In this regard, there are several studies that have investigated potential biomarkers for early detection of neurocognitive impairment in HIV infected patients (Hinkin et al., 2008; Kusao et al., 2012) while there are very limited studies that have investigated genetic factors that might be affected by HIV infection and/or cocaine abuse. Considering the close connection of DA transmission and ROS regulation by DJ1, we focused on the role of DJ1 in HIV and/or drug addiction. In this regard the present study indicated for the first time that gene expression of PARK7/DJ1 is significantly upregulated with cocaine and HIV exposure after 24 h of incubation (Figures 1 and 2). Up-regulation of PARK7/DJ1 with acute exposure of HIV-1 or cocaine indicated the active involvement of DJ1 in regulating the ROS production in neuronal cells. These results corroborated with previous studies, which have also shown the role of DJ1 in regulating the balance of ROS in the brain (Liang et al., 2007; Lopert and Patel, 2014). However, further detailed investigation on the chronic effect of HIV and cocaine exposure showed inhibitory effect on DJ1 gene in SK-N-MC. When optimized doses of HIV-1 (100 ng) and/or cocaine (1 μM) were used to treat SKN-MC cells for 24 and 72 h, there was a synergistic effect of these two factors in reducing the DJ1 gene expression due to prolong exposure of HIV-1 and cocaine (Figure 3A; *p* = 0.0006). It is noteworthy that combined effect of HIV-1 and cocaine was more significant on DJ1 expression at 72 h post incubation compared to 24 h. However, we acknowledge the fact that DJ1 expression level was variable between 24 and 72 h with HIV-1 exposure alone (Figure 3A). It has been demonstrated previously that SKN-MC get infected with both R5 and X4 strains of HIV-1 in chemokine receptors dependent and the independent pathways (Harouse et al., 1995; Alvarez Losada et al., 2002; Atluri et al., 2014). The present study also demonstrated an increase in HIV-1 replication (p24 level) in SK-N-MC up to 72 h which may have an effect in DJ1 down regulation along with cocaine which is subject to further investigation (Figure 3B). Based on these observations; further characterization was done 72 h post incubation.

The DJ1 gene down-regulation was further validated with protein profile of the DJ1 expression in SK-N-MC cell lysate as measured by Western blot and flow-cytometry, respectively (Figure 4). The western blot analysis indicated that there was significant decrease in DJ1 expression with HIV-1 exposure up to 72 h (Figure 4A). This data correlated with the HIV-1 p24 level data indicating chronic exposure of HIV significantly decreased DJ1 expression. More importantly, the combined exposures of HIV-1 and cocaine have greatly reduced the DJ1 expression indicating the direct effect as described in Figure 4A. The volumetric analysis of the protein expression has reestablished the observation of marked down-regulation of the DJ1 protein that was statistically significant (*p* < 0.0005; Figure 4B). The protein expression data was corroborated with flow cytometric analysis of SK-N-MC cells exposed to HIV-1 and/or cocaine (Figure 4C). The intracellular staining of DJ1 protein in SK-N-MC cells indicated that with exposure to HIV and cocaine the DJ1 protein level significantly decreased with 72 h, incubation. This observation goes in agreement with our western blot data suggesting chronic exposure of HIV and/or cocaine definitely reduced the DJ1 expression and possibly their functions. Previous studies have also indicated that HIV-1 protein Tat and cocaine exposure induced ROS production in the brain activating various biochemical pathways including extracellular signal-regulated kinases or ERK (Aksenov et al., 2006; Dalvi et al., 2014). At the same time it has also been demonstrated that upregulation of ROS is a hallmark of neurological disorders and DA receptor toxicity even in HIV infected patients. These observations also marked the role of ROS and DA dysregulation in HIV infected and cocaine addicted rat model (Aksenov et al., 2006; Capone et al., 2013). In the present study, we have observed that there was increased ROS production in HIV-1 and cocaine exposed SK-N-MC compared to untreated cells. The qualitative (Figure 5A) and quantitative (Figure 5B) analysis indicated that HIV-1 and cocaine exposure significantly induced the ROS production among treated cells up to 72 h. This observation explained the fact that HIV-1 and/or cocaine increased ROS production was inversely proportional to the gene and protein expression of DJ1. Thus, inhibition of DJ1 protein by HIV-1 and cocaine certainly increased the ROS production by SK-N-MC cells. However, there were no significant changes observed in 24 and 72 h ROS production indicating a chronic imbalance of ROS in these neuronal cells post exposure to HIV-1 and cocaine. The observation may corroborate the clinical scenario where de-regulation of ROS was observed in HIV infected patients with history of cocaine use (Wang et al., 2013). Thus the present study brings one step closer in understanding the molecular mechanisms of elevated ROS production in HIV infected and cocaine addicted patients. However, authors are also aware of the fact that ROS is regulated by many other cellular factors including DJ1. Being a much conserved gene and its ubiquitous presence in all human cells, it is challenging to identify the long term effect of DJ1 on ROS production in neurons *in vitro*. In this regard, *in vivo* study with DJ1 knockdown mice will be ideal to investigate specific molecular pathway involved in HIV-1 and cocaine mediated DJ1 dysregulation.

## Conclusion

HIV-associated neurological disorder among HIV-infected patients is an increasing problem in developed countries, which has significant social and economic consideration. Although, HAART has reduced the frequency of HAD among HIV-infected patients, there are still different forms of neurological disorder that persist even in treated patients with cocaine abuse. Since ROS and DA deregulation have shown to be the key cause of many neuronal dysfunctions, it has become increasingly important to understand this phenomenon especially in HIV infected and cocaine addicted patients. Research investigating the genetic of neuronal dysfunction is a new and rapidly developing field that may aid in new therapeutic strategy. However, very few studies have assessed whether genetic variations are associated with the early stage of neurological decline among HIV-infected patients and during cocaine addiction. As the era of neuronal genetics emerges, this study will bring opportunities to investigate the role of DJ1 in brain related disorders. In order to understand the relationship between HIV related neuronal pathology and recreational drug use, further studies on the genetics of the neurological dysfunction caused by HIV and cocaine are needed. Overall, this research may provide new evidence on the role of DJ1 in HIV-1 and/or cocaine induced oxidative stress regulation that may provide an important clinical implication in HIV infected patients with history of drug abuse.

### Conflict of Interest Statement

The authors declare that the research was conducted in the absence of any commercial or financial relationships that could be construed as a potential conflict of interest.
